# Structural Neurotoxic and Neuroprotective Effects of Ketamine and Esketamine in Preclinical and Human Studies: A Systematic Review

**DOI:** 10.21203/rs.3.rs-9827580/v1

**Published:** 2026-05-27

**Authors:** Tychique T. Wasolua, Tanner J. Bommersbach, Roger S. McIntyre, Taeho Greg Rhee

**Affiliations:** University of Connecticut School of Medicine; University of Wisconsin-Madison School of Medicine; University of Toronto School of Medicine; Yale School of Medicine

**Keywords:** Ketamine, esketamine, neurotoxicity, synaptic plasticity

## Abstract

Ketamine and esketamine are increasingly used as rapid-acting antidepressants, yet concerns remain regarding potential structural neurotoxicity, particularly with chronic or high-dose exposure. Despite expanding clinical use, no comprehensive systematic review has integrated neurotoxicity evidence across preclinical and human studies while formally evaluating methodological quality. Following PRISMA 2020 guidelines, we searched PubMed/Medline, Embase, PsycINFO, and Cochrane Library from inception through September 18, 2025. Eligible studies included experimental and observational designs reporting structural, molecular, or cellular neurotoxic outcomes following ketamine or esketamine exposure. Risk of bias and methodological quality were assessed. Seventy-six studies met inclusion criteria, comprising 55 animal and 21 human investigations. In preclinical models, repeated or high-dose ketamine exposure consistently produced structural neurotoxicity, including neuronal apoptosis, dendritic spine atrophy, white matter compromise, and cortical thinning, with heightened vulnerability during developmental periods. In contrast, infrequent or lower-dose therapeutic regimens (≤ 0.5 mg/kg) demonstrated minimal neurodegenerative changes, and, in stress-exposed models, paradoxically exhibited neuroprotective effects including synaptic restoration and anti-inflammatory activity. In human studies, chronic recreational ketamine use showed widespread cortical thinning and gray matter volume reductions. Conversely, therapeutic ketamine administration at controlled subanesthetic doses produced minimal structural changes. Methodological quality was medium or high among human studies, whereas preclinical studies frequently lacked clear reporting of randomization and blinding procedures. Ketamine‘s neurotoxic effects are strongly context-dependent, with risk primarily influenced by dose, cumulative exposure, developmental timing, and underlying pathophysiological state. The available evidence supports a threshold distinction between neurotoxic effects observed with chronic high-dose exposure and the relative structural safety of therapeutic dosing regimens.

## INTRODUCTION

First synthesized in 1962, racemic ketamine is a fast-acting dissociative anesthetic that has played a central role in emergency medicine, procedural sedation, and analgesia for decades.^1^ More recently, ketamine has gained substantial attention in psychiatry following evidence of its rapid antidepressant effects in treatment-resistant depression (TRD).^2^ These findings led to the regulatory approval of intranasal esketamine, the *S*-enantiomer of ketamine by the U.S. Food and Drug Administration (FDA) in 2019 and the European Medicines Agency (EMA) in 2020, marking the introduction of the first antidepressant with a novel mechanism of action in decades.^3^

The expansion of ketamine into psychiatric practice has occurred alongside marked regulatory divergence between formulations. Esketamine is dispensed exclusively under a Risk Evaluation and Mitigation Strategy (REMS), which mandates supervised administration, post-dose monitoring, and restrictions on dosing frequency. In contrast, racemic ketamine remains unapproved for psychiatric indications and is prescribed off-label without standardized oversight or harmonized safety protocols.^4^ Consequently, clinical practice patterns vary substantially, and real-world treatment often exceeds the dosing schedules and cumulative exposure limits studied in controlled trials.^5^ These differences raise important public health concerns, particularly because high-dose or repeated exposures may carry neurobiological risks that remain incompletely characterized.^5–8^

Biological mechanisms further underscore the need for careful evaluation. Ketamine primarily acts as a noncompetitive N-methyl-D-aspartate receptor (NMDAR) antagonist, blocking ion flux through the receptor’s open channel.^9^ NMDARs play essential roles in synaptic plasticity, including long-term potentiation and depression, which underlie learning and memory.^10^ Beyond synaptic signaling, NMDAR activation is critical for neuronal survival, dendritic maturation, and circuit refinement, particularly during sensitive developmental windows. These receptors regulate calcium-dependent intracellular cascades that influence gene transcription, protein synthesis, and structural synaptic remodeling.^11^ Pharmacologic disruption of these pathways through sustained or repeated pharmacologic NMDAR blockade therefore raises plausible concerns about neuronal injury, altered dendritic architecture, and long-term neurodevelopmental effects. Such concerns are reinforced by findings with other high-affinity NMDA antagonists, such as MK-801 (dizocilpine), which induce acute vacuolar neurodegeneration in preclinical models.

Despite growing attention to ketamine’s potential neurotoxic effects, the existing literature remains fragmented. Most prior reviews have been narrative rather than systematic, limiting their capacity to inform evidence-based practice and regulatory guidance.^4^ No comprehensive systematic review has integrated structural neurotoxicity outcomes across both preclinical and human studies while rigorously evaluating methodological quality and risk of bias. Although recent scoping efforts have catalogued relevant studies,^4^ they did not assess study quality, evaluate bias, or synthesize the strength and consistency of evidence using validated frameworks.

To address these gaps, this systematic review aims to comprehensively evaluate and appraise evidence for ketamine-associated structural neurotoxicity across preclinical models and human studies, with particular emphasis on methodological quality, risk of bias, and the moderating factors that may shape neurotoxic risk.

## METHODS

This systematic review was conducted in accordance with PRISMA 2020 guidelines. The review question and search strategy were developed by the primary authors (T.T.W., T.G.R): *Does exposure to racemic ketamine or esketamine in human or preclinical studies produce measurable structural, molecular, or cellular neurotoxic effects in the brain?* Using a PEcO (population, exposure, comparison, outcome) framework, we examined associations between ketamine or esketamine exposure and neurotoxic outcomes across experimental and observational study designs. Eligible studies reported neurobiological or structural indicators of neurotoxicity following ketamine or esketamine exposure. For the purpose of this review, neurotoxicity was operationally defined to include not only overt cellular injury (e.g., apoptosis, necrosis), but also maladaptive changes in synaptic structures, function or connectivity that may precede or occur independently of frank neural death. This broader definition reflects the mechanistic understanding that NMDA receptor antagonism can disrupt synaptic plasticity and network integrity without necessarily causing immediate cell loss. Studies were excluded if they lacked endpoints relevant to these domains, examined non-ketamine NMDA antagonists, involved polysubstance exposure where ketamine effects could not be isolated, or were not published in English. Full eligibility criteria are detailed in **Supplemental Table 1**.

### Search strategy and study selection

A comprehensive literature search was conducted using Ovid, covering PubMed/Medline, Embase, PsycINFO, and the Cochrane Library, from database inception through September 18, 2025. Search terms are detailed in **Supplemental Table 2**. Records were imported into EndNote for deduplication and screening. Titles and abstracts were screened for relevance, followed by full-text review of potentially eligible articles.

**Figure 1.**
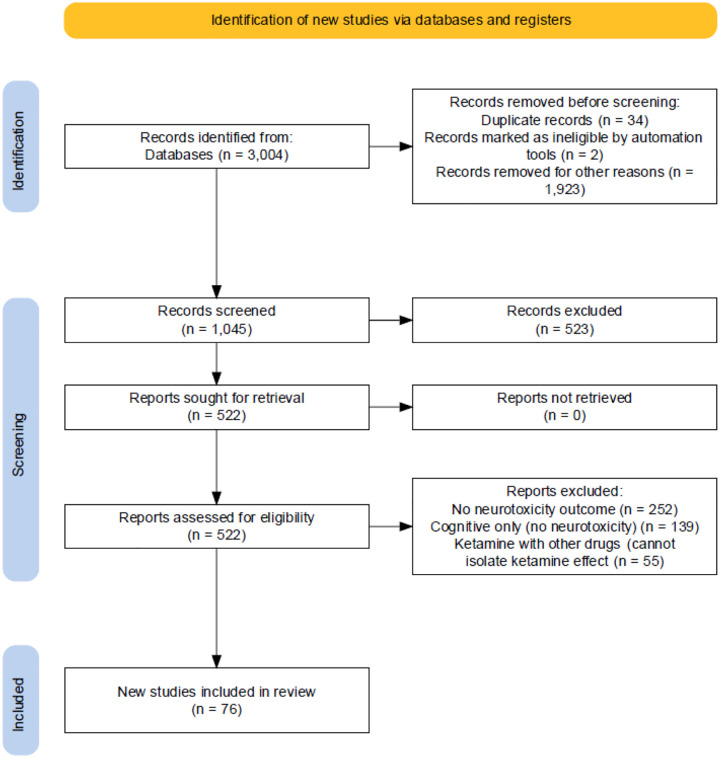
PRISMA 2020 flow diagram of study selection **Note:** Study attrition diagram based on PRISMA 2020 guidelines.

The search identified 3,004 records, of which 76 met inclusion criteria and were included in the final synthesis, comprising 55 animal studies and 21 human or in vitro investigations. Study selection is summarized in the PRISMA 2020 flow diagram (Fig. 1; **Supplemental Table 3**).

#### Data extraction

Data were extracted by one reviewer (T.T.W.) using standardized extraction forms, with human and animal studies analyzed separately. Given regulatory and clinical relevance, we prospectively coded studies by ketamine formulation: racemic ketamine, *S*-ketamine (*S*-enantiomer), esketamine (pharmaceutical-grade *S*-enantiomer), or ketamine metabolites including (2R,6R)-hydroxynorketamine. Throughout this review, ‘esketamine’ and ‘*S*-ketamine’ refer to the same compound, the *S*-enantiomer of ketamine, and are used according to the terminology employed in the original studies.

#### Animal studies

For animal studies, extracted variables included species, developmental stage, ketamine formulation, dosing regimen, exposure route and duration, histopathological findings, molecular or biochemical markers of injury, neuroimaging results when applicable, and relevant behavioral correlates (Table 3).

#### Human studies

For human studies, extracted information included study design, setting, participant characteristics, ketamine formulation, route of administration, dosage, exposure duration, neurotoxic outcomes (e.g., structural MRI findings, volumetric changes, white matter alterations, neural injury biomarkers), and adverse neurological events. Cognitive outcomes were extracted only when they were mechanistically or anatomically linked to underlying neurotoxicity. Extracted data are summarized in Table 1.

#### Risk of bias assessment and certainty of evidence

Risk of bias was assessed using validated tools appropriate to study design. Randomized controlled trials (RCTs) were assessed using the Cochrane Risk of Bias 2 tool (**Supplemental Fig. 1**).^12^ Observational human studies were assessed using the Newcastle Ottawa Scale (**Supplemental Table 4**). Preclinical animal studies were assessed using the SYRCLE Risk of Bias tool (**Supplemental Table 5** and **Supplemental Fig. 2**).^13^ The certainty of evidence for human studies was evaluated using the Oxford Centre for Evidence-Based Medicine 2011 Levels of Evidence.

### Data synthesis

Due to substantial heterogeneity in species, dosing regimens, exposure durations, and neurobiological endpoints, quantitative meta-analysis was not feasible. Thus, findings were synthesized narratively and organized into two major domains: preclinical and human neurotoxicity evidence, with results grouped by neurobiological outcome.

## RESULTS

### Study characteristics

Of 3,004 articles identified, 76 met inclusion criteria (Fig. 1). These included 56 animal studies, 19 human studies, and one *in vitro* study using a human cell line. Study characteristics are summarized in Table 2 and Table 3.

### Part 1. Animal models

The preclinical literature was dominated by rodent models, including rats (n = 29) and mice (n = 23), with a smaller number of studies in Cynomolgus monkeys and Mongolian gerbils.^14–17^ Study characteristics and dosing parameters are detailed in Table 3. Of the 56 preclinical studies, the majority (n = 48) employed racemic ketamine, with a smaller subset investigating *S*-ketamine (n = 4), esketamine (n = 2) or the metabolite (2R,6R)-hydroxynorketamine (n = 2). No preclinical studies directly compared neurotoxicity profiles between enantiomers. Methodologically, animal studies employed multi-modal approaches. Behavioral outcomes were commonly assessed using paradigms such as the forced swim test (FST) to measure depressive-like states and cognitive function and correlated with structural and molecular changes quantified via immunohistochemistry (e.g., Ki-67, NeuN, and Iba1), Western blotting for neurotrophic factors (e.g., BDNF and PSD-95), and Golgi-Cox staining for dendritic spine morphology.^18,19^ Functional synaptic plasticity was assessed in 21 studies through electrophysiological recordings,^17,20–39^ while a smaller subset utilized neuroimaging techniques such as fMRI or Diffusion Tensor Imaging (DTI) to map functional connectivity and white matter integrity.^15,40–42^ Collectively, these studies focused on the neurobiological consequences of repeated or high-dose ketamine exposure, particularly during development.

### Apoptotic neurodegeneration and cell loss through histological staining

Three animal studies reported increased apoptotic markers and neuronal loss following chronic or prenatal ketamine exposure at dose ranging from 1mg/kg IV daily for 6 month (adolescent primates) to 60 + 20mg/kg maternal dosing (prenatal rats).^14,43,44^ In adolescent cynomolgus monkeys receiving ketamine 1 mg/kg IV daily for 6 months, TUNEL-positive cells in the prefrontal cortex increased significantly relative to controls (p < 0.05), accompanied by elevations in Bax and caspase-3 expression.^14^ Similarly, prenatal ketamine exposure in rats (40–60 mg/kg single maternal dose) resulted in persistent reductions in hippocampal CA3 cell density and decreased proliferative cells in the dentate gyrus and subventricular zone.^45^ In adolescent rats, repeated daily administration of 20–30 mg/kg for 21 days increased TUNEL-positive apoptotic cells in the prefrontal cortex. In contrast, single-dose or short-term ketamine (12.5 mg/kg) exposure in adult rats showed no microscopic neuronal necrosis or Olney’s lesions.^46^

### Dendritic spine atrophy and synaptic protein reduction through morphometric analysis

Ten animal studies demonstrated context-dependent effects of ketamine on dendritic structure.^22,32,37,47–53^ In stress-based and genetic depression models, low dose ketamine (10 mg/kg single dose) reversed dendritic atrophy and restored spine density and synaptic protein expression (PSD-95, GluA1). Specifically, single dose *S*-ketamine (10 mg/kg) produced significant increases in dendritic spine density in the medial prefrontal cortex (*p* < 0.05) and single-dose racemic ketamine (10 mg/kg) full restoration of hippocampal dendritic length in chronic mild stress models (*p* < 0.05).^22,47,49–53^ Prophylactic ketamine (3 mg/kg single dose) administered before chronic stress induced significant changes in dendritic spine density and morphology in depression-related brain regions.

In contrast, chronic administration in healthy adult mice (20–30 mg/kg daily for 14–28 days) reduced CA1 spine density and downregulated PSD-95 (*p* < 0.05), while repeated neonatal exposure (20 mg/kg three times daily for 3 days) decreased dendritic branch number (*p* < 0.01), total dendritic length (*p* < 0.01), and spine density (*p* < 0.01) in the CA1 region.^32,37,43^ Ultrastructural analyses further revealed thinning of the postsynaptic density and widening of the synaptic cleft (*p* < 0.01).^37^ Repeated ketamine in adolescent mice (20 mg/kg twice daily for 14 days) similarly reduced PSD-95 expression in the hippocampal CA1 region.^54^

### Functional synaptic plasticity, transmission, and network dynamics

Eighteen studies assessed synaptic plasticity and transmission.^20–28,31,32,34–39,55^ Across these animal studies, exposure to racemic ketamine or esketamine modulated specific physiological metrics, including Hebbian long-term potentiation (LTP), depression (LTD) and homeostatic scaling of spontaneous excitatory postsynaptic currents (sEPSCs) in a context-dependent manner. Subanesthetic ketamine (5–10 mg/kg) consistently restored long-term potentiation and synaptic transmission impaired by stress models, while in healthy tissue, low-dose ketamine (110 μM in vitro; 10 mg/kg in vivo) selectively inhibited NMDAR-dependent long-term depression without affecting long-term potentiation.^17,35,38,39^ In a social isolation model, ketamine (5 mg/kg) rescued long-term potentiation deficits (*p* < 0.05).^39^ In contrast, repeated high-dose ketamine in healthy mice reduced synaptic transmission (*p* < 0.001) and long-term potentiation magnitude (*p* < 0.01).^31^

Five additional studies examined electrophysiological oscillations and calcium signaling, with distinct profiles for acute versus chronic ketamine exposure.^16,26,56–59^ Acute racemic ketamine exposure (single dose of 2.5–15 mg/kg) consistently increased gamma activity, whereas chronic exposure (2.5–10 mg/kg, once daily for 4 weeks) reduced gamma coherence and shifted activity toward slower alpha rhythms.^16,26,57,58^ In a mouse model of bipolar disorder, calcium imaging revealed significant hypoactivity in the temporal cortex (*p* < 0.001) and prefrontal cortex following single dose (30 mg/kg) of ketamine combined with stress exposure.^60^

### Functional connectivity, white matter integrity, and neuroinflammatory signaling

Animal neuroimaging studies demonstrated dose- and exposure-dependent network reorganization.^15,40–42^ Acute low-dose *S*-ketamine (10 mg/kg) increased connectivity in prefrontal and reward circuits, often normalizing stress-induced dysconnectivity.^41^ In contrast, chronic ketamine exposure in adolescent primates (1 mg/kg IV daily for 6 months) reduced activation in dopaminergic midbrain regions.^61^ In neonatal rats, repeated high-dose esketamine (25–50 mg/kg daily for 3 days) produced dose-dependent white matter damage in the corpus callosum detectable by diffusion tensor imaging, with the higher dose (50 mg/kg) causing more severe microstructural abnormalities,^62^ while chronic or high-dose exposure in developmental models compromised white matter integrity and reduced activation in dopaminergic midbrain regions.^15,40–42^

Nine animal studies examined neuroinflammatory and oxidative pathways.^18,35,47,52,63–67^ In stress models, single dose (5 or 10 mg/kg i.p.) of racemic ketamine dose dependently attenuate elevation in pro-inflammatory cytokines and a single dose of esketamine (10 mg/kg i.p.) reduced oxidative stress markers, while *S*-ketamine (10 mg/kg i.p.) suppressed NLRP3 inflammasome activation, and attenuated astrocytic pyroptosis.^35,47,52,63,65^

In contrast, chronic high-dose ketamine exposure (20 mg/kg, 14 days) in healthy mice increased oxidative stress markers and reduced antioxidant enzyme activity (*p* < 0.001).^18^

### Part 2. Human studies

Nineteen human studies were included, comprising 18 clinical or observational investigations and one pharmacovigilance analysis using the FDA Adverse Event Reporting System.^68–85^ Of the clinical studies, 13 (72%) were prospective, including six RCTs,^68,70,71,75–77^ with the remainder consisting of cohort, case–control, retrospective,^72–74,78,79,81^ and three cross-sectional investigations primarily examining chronic recreational ketamine use.^83–85^ Regarding formulation, 11 studies (58%) investigated racemic ketamine, 5 studies (26%) examined esketamine specifically, and 3 studies (16%) used S-ketamine or mixed formulations. Excluding the pharmacovigilance study, the 18 clinical investigations included 1,184 participants (686 ketamine/esketamine-exposed; 498 controls). Participant ages ranged from 22.1 to 67.6 years.

### Ketamine treatment characteristics

Across RCTs, ketamine was most commonly administered as a single intravenous infusion for antidepressant or neurocognitive studies. Five trials used 0.5 mg/kg over 40 minutes, one cross-over trial used *S*-ketamine 0.25 mg/kg over 40 minutes,^68,70,71,76,78^ and one trial administered intranasal esketamine (56 mg).^77^ Another RCT used intravenous esketamine at 0.2 or 0.5 mg/kg, and one study employed a target-controlled infusion maintaining plasma levels at 100 ng/mL.^75,80^ In the 6 other prospective and naturalistic cohort studies, dosing focused on serial administration. Esketamine (0.25–0.5 mg/kg) was administered three times weekly for two weeks in three studies,^72,73,78^ while racemic ketamine (0.5 mg/kg) was given four times over two weeks in treatment-resistant depression cohorts.^74,79,81^ Recreational user studies reported substantially higher self-administered doses, including a mean intranasal dose of 0.14 g (~ 2 mg/kg) per session, often in the context of polydrug use.^82^

### Neuroimaging of structural and functional connectivity

Fourteen studies employed neuroimaging modalities to assess the structural and functional sequelae of ketamine exposure, including fMRI (n = 8), structural MRI (n = 5), and diffusion tensor imaging (DTI).^70,73,74,76–81,83,85^

Chronic recreational ketamine use was consistently associated with structural abnormalities, including widespread cortical thinning and gray matter volume reductions, particularly in frontal and parietal regions (p < 0.01).^83,85^ In therapeutic contexts, structural findings were mixed. Small reductions in dorsolateral prefrontal cortex volume were reported following a single infusion in one study,^71^ while others reported stable hippocampal volumes, preserved white matter integrity, or transient regional volume increases.^73,77,81^

Functionally, acute exposure of subanesthetic esketamine (e.g., 0.25 mg/kg IV) or racemic ketamine in healthy volunteers consistently reduced resting-state network connectivity in healthy volunteers.^70,76,80^ In depressed populations, serial therapeutic treatments with racemic ketamine (0.5 mg/kg) or esketamine (0.25 mg/kg) induced functional reorganization within habenular, hippocampal, and striatal networks.^68,72,79^ Furthermore, task-based functional MRI following repeated racemic ketamine exposure (0.5 mg/kg IV) demonstrated altered amygdala reactivity and prefrontal activity.^74^

### Molecular biomarkers and physiological responses

Two studies evaluated serum biomarkers. High-dose esketamine (0.5 mg/kg) significantly reduced S100β, decreased IL-6, and increased BDNF in surgical patients, whereas low-dose esketamine (0.2 mg/kg) produced no significant changes in S100β or BDNF.^75^ In chronic recreational users, serum BDNF levels were significantly elevated, while NGF levels did not differ from controls.^84^

### Dose–frequency distribution of structural neurotoxicity

**Figure 2.**
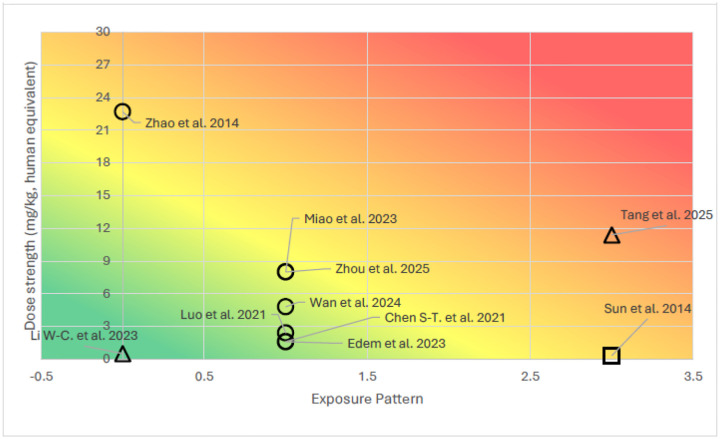
Dose–frequency map of structural neurotoxicity associated with ketamine exposure **Note**: Each point represents a study demonstrating structural neurotoxic outcomes, plotted by human-equivalent dose strength (y-axis) and exposure frequency category (x-axis). Marker shape denotes species (rodent [circle], nonhuman primate [square], human [triangle]). Exposure categories: 0 = single dose; 1 = intermittent, <2 months; 2 = frequent, ≥2 months; 3 = daily, ≥3 months. Marker shape denotes species (rodent [circle], nonhuman primate [square], human [triangle]). The background gradient illustrates increasing likelihood of neurotoxicity with higher dose and greater exposure frequency.

To visualize the relationship between ketamine exposure patterns and structural neurotoxic outcomes, we constructed a dose–frequency map integrating all studies demonstrating morphological or histopathological injury across preclinical and human populations (Fig. 2). Neurotoxic effects clustered at higher dose strengths and increased exposure frequencies, with moderate to severe injury predominantly observed in repeated or chronic exposure paradigms. Developmental and neonatal exposures at moderate-to-high doses were consistently associated with dendritic abnormalities, synaptic damage, and neuronal loss in preclinical models, while prolonged daily exposure in nonhuman primates and chronic high-dose recreational use in humans were associated with marked cortical injury. In contrast, isolated low-frequency exposures were associated with minimal or no detectable structural changes.

### Risk of bias and methodological quality

Methodological quality varied across study designs. Most RCTs demonstrated low risk of bias, with isolated concerns related to randomization or reporting (**Supplemental Fig. 1**). Observational human studies were predominantly of high quality on the Newcastle–Ottawa Scale (**Supplemental Table 4**). Oxford Centre for Evidence-Based Medicine Levels of Evidence classifications are provided in Table 2. In contrast, preclinical studies exhibited substantial uncertainty across the SYRCLE domains due to incomplete reporting of randomization, allocation concealment, blinding, and outcome assessment procedures (**Supplemental Table 5**; **Supplemental Fig. 2**).

## DISCUSSION

This systematic review synthesizes evidence from 76 preclinical and human studies examining the neurotoxic effects of ketamine and esketamine across diverse exposure paradigms, developmental stages, and neurobiological outcomes. Across preclinical studies, repeated or high-dose ketamine exposure consistently produced structural neurotoxicity characterized by neuronal apoptosis, dendritic spine atrophy, white matter disruption, and cortical thinning, especially when administered during vulnerable developmental periods. In contrast, infrequent or low-dose therapeutic regimens (e.g., < 0.5 mg/kg in adult animals) did not elicit overt neurodegenerative changes in rodent or primate brains, with most controlled studies reporting minimal or transient structural changes, and in stress-exposed models, ketamine frequently exhibited neuroprotective effects, including synaptic restoration and anti-inflammatory activity.

### Consistency and divergence with prior studies

While providing a more systematic perspective, our findings align with prior narrative reviews describing ketamine’s *context-dependent* neurobiological effects, in which the drug can produce both neurotoxic and neuroprotective effects depending on dose, exposure pattern, and biological state.^86–88^ This duality is affirmed by our systematic synthesis.

Our findings align with established neurodevelopmental toxicology showing that NMDAR antagonism during critical developmental windows induces apoptotic neurodegeneration, likely through disruption of activity-dependent survival signaling.^89,90^ Neonatal rodent studies in our review demonstrated increased apoptotic markers and reduced neuronal density following repeated ketamine exposure, particularly in hippocampal and cortical regions,^43,91,92^ while chronic exposure in adolescent nonhuman primates extended these concerns to phylogenetically closer species.^14^

Our findings are also concordant with the recent narrative review by Li et al. (2025), which reported that high-dose ketamine induces neurodegeneration in animal models, whereas intermittent low-dose exposure does not produce overt histopathological changes,^4^ and that long-term esketamine treatment in depressed patients does not appear to impair cognition a point our review confirms with additional data from newer trials.^4^ Importantly, our work builds upon these prior reviews by systematically evaluating study quality and risk of bias across both preclinical and human studies.

Our findings are consistent with the synaptogenic hypothesis of ketamine’s antidepressant mechanism, whereby NMDAR blockade triggers a cascade involving AMPAR activation, BDNF release, and leading to dendritic spine formation and synaptic strengthening.^93^ However, key components of this model remain debated: mTOR activation has not been consistently replicated as essential for ketamine’s antidepressant effects, including in a clinical study that found no association between mTOR signaling and treatment response.^94^ Furthermore, recent evidence suggests that dendritic spine formation may contribute to sustained, rather than rapid, antidepressant effects, as behavioral improvements precede structural spine changes.^95^ In stress-exposed models, seven of ten preclinical studies demonstrated increased spine density, dendritic length, synaptic protein expression, and restoration of impaired long-term potentiation, with temporal correlation to antidepressant-like behavioral effects.^17,29,35,47,52,53,66^ These effects occurred specifically in models with baseline synaptic deficits, suggesting that ketamine’s neuroplastic actions require a pathological substrate. In contrast, administration of equivalent or higher doses to healthy animals without prior stress consistently reduced spine density, downregulated synaptic proteins, and induced ultrastructural synaptic damage.^32,37,43^ This bidirectional pattern suggests that NMDAR antagonism interacts with baseline neuroplasticity state, potentially restoring plasticity in stress-primed circuits while disrupting activity-dependent stabilization in homeostatic conditions.^96,97^ This interpretation is consistent with selective inhibition of NMDAR-dependent long-term depression without impairment of long-term potentiation in healthy tissue and aligns with the inverted-U relationship of glutamatergic signaling, whereby both excessive activation and profound suppression are injurious.^98,99^ Together, these findings indicate that dose, cumulative exposure, and underlying pathophysiology are key determinants of neurotoxic risk.

Within the clinical studies included in this review, frequency-dependent structural data are largely clustered at two extremes. Short-term therapeutic protocols involving racemic ketamine or esketamine (single dose or 4–6 sessions over 2 weeks) generally reported stable white matter and hippocampal volumes, though one study reported a small but significant gray matter volumetric reduction in the right dorsolateral prefrontal cortex following a single dose of racemic ketamine.^71,73,100^ In contrast, chronic recreational racemic ketamine use was consistently associated with widespread cortical thinning and gray matter reductions.^101,102^ Preclinical data offer more granular insight into this dose-time relationship: daily administration of racemic ketamine (30 mg/kg) in mice for up to 10 days produced no alterations in long-term potentiation (LTP) or basal fEPSP slopes, whereas 28 continuous daily doses impaired CA1 LTP induction and reduced basal synaptic transmission alongside synaptic protein loss; conversely, spaced intermittent dosing of racemic ketamine (e.g., 8 doses over 4 weeks) produced no working memory or synaptic transmission deficits.^103,104^ These findings would suggest that dosing interval may be as important as cumulative exposure. Furthermore, while many preclinical studies employed higher doses designed to model schizophrenia or achieve anesthesia, these paradigms remain informative, consistently producing structural neurotoxicity unlike low-dose regimens, possibly delineating an upper threshold at which adaptive neuroplasticity shifts into structural damage. Therefore, it is important to recognize that the majority of preclinical neurotoxicity findings are not directly generalizable to therapeutic antidepressant dosing, which operates well below these thresholds.

### Mechanistic interpretation and biological plausibility

#### NMDAR blockade and excitatory-inhibitory imbalance

The structural neurotoxicity observed with chronic high-dose ketamine exposure likely reflects sustained disruption of excitatory–inhibitory circuit balance downstream of NMDA receptor antagonism. Ketamine preferentially inhibits NMDA receptors on GABAergic interneurons, particularly parvalbumin-positive interneurons, which play a central role in regulating cortical excitation.^105^ Suppression of these inhibitory cells leads to disinhibition of pyramidal neurons and a compensatory increase in glutamate release, as reflected by elevated extracellular glutamate levels and increased cortical gamma activity following ketamine administration.^15,57,59,84^ While brief glutamate elevations may support adaptive synaptic plasticity and neurotrophic signaling associated with antidepressant effects, repeated or prolonged disinhibition may overwhelm regulatory mechanisms, promoting excitotoxic stress through calcium overload,, mitochondrial dysfunction, and apoptotic signaling. This mechanistic hypothesis aligns with electrophysiological findings in our review, in which acute ketamine consistently enhanced gamma oscillations, reflecting synchronized fast-spiking interneuron activity and cortical hyperexcitability, whereas chronic exposure produced alpha-band slowing and cortical hypoactivity, potentially indicating compensatory downregulation or interneuron dysfunction.^16,57,59^ This transition from acute hyperexcitability to chronic hypoactivity mirrors the temporal progression from initial glutamate surge to eventual synaptic depression observed in other excitotoxic paradigms, supporting a biphasic injury model.^106,107^

#### Developmental vulnerability and critical period sensitivity

The heightened neurotoxic susceptibility during prenatal and neonatal periods reflects the essential role of NMDAR signaling in developmental processes including neuronal migration, dendritic arborization, synapse formation, and activity-dependent circuit refinement. NMDA receptors are critical regulators of synaptic plasticity and neuronal survival; their activation permits calcium influx that drives gene expression, trophic factor release, and normal dendritic development.^108,109^ Blocking these receptors, especially during development, may disrupt activity-dependent neurodevelopmental processes, leading to excessive neuronal elimination beyond physiological programmed cell death. Moreover, GABAergic interneurons mature more slowly than pyramidal neurons, with key inhibitory features such as parvalbumin expression developing after birth.^110,111^ Interference with NMDA receptor dependent maturation during this sensitive period may therefore lead to lasting disruption of excitatory-inhibitory balance.

#### White matter vulnerability and oligodendrocyte sensitivity

White matter abnormalities observed with chronic ketamine exposure, including reduced fractional anisotropy and altered diffusion metrics, may reflect oligodendrocyte injury or myelination deficits. Oligodendrocytes express functional NMDARs that regulate survival, differentiation, and myelin synthesis; NMDAR antagonism can suppress oligodendrocyte precursor proliferation and impair myelin formation.^112^ Additionally, white matter is particularly vulnerable to oxidative stress due to high lipid content and metabolic demands of myelin maintenance.^113,114^ The combination of direct NMDAR blockade on oligodendrocytes and indirect oxidative injury from chronic high-dose exposure may synergistically compromise white matter integrity, producing microstructural abnormalities detectable by neuroimaging. The clinical significance of white matter changes in chronic users remains incompletely characterized, but the anatomic distribution, affecting corpus callosum, cingulum, and superior longitudinal fasciculus,^40,73^ overlaps with tracts implicated in executive function and memory, domains commonly impaired in chronic ketamine users.^115–117^

#### Pharmacological distinctions between racemic ketamine and esketamine

Although the title of this review encompasses both ketamine and esketamine, it is important to note that preclinical evidence derives predominantly from racemic ketamine (85% of animal studies), with only two studies employing pharmaceutical grade esketamine. This imbalance warrants caution when extrapolating preclinical neurotoxicity findings to esketamine specifically. Esketamine exhibits approximately 3–4 fold higher affinity for the NMDA receptor than R-ketamine and demonstrates distinct pharmacokinetic properties, including faster clearance and different metabolite profiles. Clinically, intranasal esketamine is administered under controlled, intermittent protocols mandated by REMS, whereas racemic ketamine is prescribed off-label without standardized oversight, potentially resulting in substantially different cumulative exposure patterns. Whether the neurotoxicity thresholds established primarily from racemic ketamine studies apply equivalently to esketamine remains uncertain. The available human esketamine data, though limited to 5 studies in this review, have not identified structural neurotoxicity at therapeutic doses, but longer-term follow-up data are needed before definitive conclusions can be drawn.

#### Clinical and regulatory implications

The evidence synthesized here supports a clear threshold distinction between neurotoxic chronic misuse and therapeutic safety profiles. Controlled subanesthetic administration (≤ 0.5 mg/kg) over limited durations appears neurobiologically distinct from chronic recreational use characterized by higher per-session doses, frequent repetition, and substantial cumulative exposure. Our synthesis found no evidence of structural neurotoxicity in patients receiving esketamine at controlled therapeutic doses across the available study durations, which were predominantly limited to single infusion, or 2–4 weeks protocols. Critically however, the evidence base does not extend to the longer treatment duration increasingly employed in clinical practice, and extrapolation of short-term safety data to maintenance therapy remains unsubstantiated. Clinically, the REMS framework mandating supervised administration, post-dose monitoring, and strict limits on dose and frequency may mitigate neurotoxic risk by maintaining exposure within parameters shown here to be structurally safe.^118^ Notably, emerging real-world evidence from post-marketing surveillance and naturalistic cohort studies has reported favorable safety and tolerability profiles for intranasal esketamine in clinically complex, treatment-resistant populations, with reassuring long-term safety outcomes, and pharmacovigilance analyses using FAERS databases confirming a relatively favorable urological and general tolerability profile.^119–121^ Importantly, these reassuring findings were obtained under the condition imposed by REMS framework. Whether this similar safety would be observed with unsupervised or escalating off-label use remains unknown, and caution is warranted against extrapolating safety data from controlled clinical use to unsupervised or escalating off-label exposure.

### Strength and limitations

This review employed a rigorous systematic methodology aligned with PRISMA 2020 guidelines, including comprehensive database searching across PubMed/Medline, Embase, PsycINFO, and Cochrane Library (spanning diverse species, exposure regimens, and outcome measures) and synthesizing a large body of evidence that was previously fragmented. The application of quality assessment frameworks enhances methodological transparency and facilitates interpretations. The use of validated, design-specific risk-of-bias tools strengthens the reliability of quality appraisal. The explicit PEcO framework provided clear operational definitions for exposure, population, and outcomes, minimizing selection ambiguity. Importantly, to our knowledge, this review is the first to systematically synthesize structural neurotoxicity outcomes across both preclinical and clinical populations while formally evaluating methodological quality and risk of bias.

Regardless of the strength of this review, several methodological limitations warrant consideration. First, the majority of therapeutic human studies included in this review enrolled individuals with treatment-resistant depression, a population known to exhibit baseline differences in brain structure and/or functional connectivity independent of pharmacologic exposure.^122^ These pre-existing neurobiological abnormalities may complicate attribution of observed neuroimaging findings specifically to ketamine or esketamine, as changes may reflect illness-related pathology, treatment response, or their interaction rather than drug-induced neurotoxicity alone.

Second, the preclinical literature is dominated by repeated or high-dose ketamine, often at doses designed to model schizophrenia (20–30 mg/kg) or achieve anesthesia (≥ 50 mg/kg), which exceed clinically relevant exposures, potentially overestimating neurotoxic risk at therapeutic doses.^22,37,40,44,66,123^ Whereas the therapeutic human literature derives almost exclusively from acute or short-term administration protocols involving single infusions or 2–4 week serial treatments. This asymmetry means that preclinical data can inform the biological mechanisms and upper bounds of neurotoxic risk, but cannot directly validate the safety of prolonged maintenance ketamine therapy in humans. Third, the human evidence base for chronic recreational use is compromised by substantial confounding from polydrug use, particularly concurrent alcohol, cannabis, and amphetamine consumption, making isolated ketamine effects difficult to disentangle. Finally, we are limited by how we can detect neurotoxicity. For example, subtle cellular changes (e.g., synaptic pruning and mild apoptosis) in human patients could go undetected if they do not produce obvious imaging changes or clinical symptoms. Thus, an absence of evidence of harm does not mean an absence of effect; it simply indicates that any potential structural changes were below our current detection thresholds.

### Conclusions

This systematic review demonstrates that racemic ketamine and esketamine’s neurotoxic and neuroplastic effects are highly context-dependent, shaped by dose, cumulative exposure, developmental timing, and underlying neurobiological vulnerability. Evidence consistently links chronic high-dose recreational use of racemic ketamine to structural neurotoxicity, whereas controlled therapeutic administration at subanesthetic racemic ketamine or esketamine doses is associated with minimal structural change and, in stress-exposed models, potential neuroprotective benefits. However, because the current clinical literature regarding chronic long-term exposure and recreational abuse exclusively evaluates racemic ketamine, the available primary data do not support fully extrapolating these long-term structural toxicity or safety profiles to esketamine. Simultaneously, the off-label psychiatric use of racemic ketamine currently lacks standardized oversight, raising concerns that real-world treatments may exceed the exposure limits evaluated in controlled trials. As clinical use of both formulations continues to expand, long-term prospective monitoring and clearly defined exposure thresholds will be essential to ensure that real-world treatment patterns remain within established safety thresholds, and do not approximate the exposure levels associated with neurotoxicity.

## Supplementary Material

This is a list of supplementary files associated with this preprint. Click to download.

- NPP260743SupplementalmaterialsFinal.docx

## Figures and Tables

**Table 1:** Characteristics and Neurotoxic Outcomes of Included Human Studies

Author	Year	Population Size	Study Design	Dosing Regimen	Duration of Administration	Neurotoxic/Neuroimaging outcome	Key Findings
Chen et al.	2020	N = 96 (48 TRD, 48 HC)	Double-blind, placebo-controlled	0.5 mg/kg or 0.2 mg/kg	Single infusion (40 min)	Resting-state fMRI (Functional Connectivity)	Responders (46.9%) showed lower pre-treatment frontostriatal connectivity than non-responders.
Curran & Morgan	2000	N = 39 (20 users, 19 controls)	Parallel group, longitudinal (Day 0 & Day 3)	Mean ~ 0.14g (range 0.06–0.5g)	Single night usage	Cognitive testing / Dissociation scales	Acute impairment in working/episodic/semantic memory. Day 3: Persistent semantic memory impairment and dissociative symptoms.
Guo et al.	2022	N = 5,592,554 (993 ketamine neuro-AEs)	Disproportionality analysis (FAERS)	Various	2019–2021 (reporting period)	Neurological Adverse Events (AEs)	Sedation, dizziness, and headache were most common. Serious AEs associated with higher doses and polypharmacy.
Hung et al.	2020	N = 53 (34 users, 19 controls)	Observational	Avg duration 4.82 years	Chronic (Adolescent vs adult onset)	Structural MRI (GMV) & Functional Connectivity	Users had lower GMV in left precuneus, right insula, left DLPFC. Adolescent-onset users had larger decrease in precuneus GMV.
Krystal et al.	2003	N = 36	Retrospective comparison	0.7 to 2.8 mg/kg (Avg 1.31)	Single bolus during ECT	Seizure duration / Reorientation time	Ketamine increased seizure duration compared to methohexital but did not significantly improve reorientation time.
Li, W.-C. et al.	2023	N = 48 (24 Ket, 24 Midazolam)	Double-blind, active-controlled	0.5 mg/kg Ket vs 0.045 mg/kg Midazolam	Single infusion	Structural MRI (VBM) / Glucose metabolism	Ketamine group showed small but significant GM volume reduction in right DLPFC on Day 3 postinfusion vs Midazolam.
Liebe et al.	2018	N = 59 (29 Ket, 30 Placebo)	Double-blind, placebo-controlled	0.5 mg/kg	Single infusion (40 min)	rs-fMRI (Locus Coeruleus connectivity)	Ketamine reduced functional connectivity between the Locus Coeruleus and the thalamus.
Liu (X) et al.	2025	N = 40 (20 MDD, 20 HC)	Longitudinal (2 weeks)	0.25 mg/kg	3x/week for 2 weeks	DTI / TBSS (White Matter Integrity)	MDD patients had widespread white matter deficits. Esketamine improved symptoms but did not reverse white matter microstructural damage.
Liu (X) et al.	2025	N = 29	Longitudinal (Pre vs Post)	0.25 mg/kg	3x/week for 2 weeks	rs-fMRI (Hippocampal Subregions)	Significant increase in FC between right caudal hippocampus and left cerebellum/precuneus/MTG post-treatment.
Loureiro et al.	2020	N = 76 (27 Ket, 17 ECT, 32 HC)	Longitudinal (Pre vs Post treatment)	0.5 mg/kg	4 infusions over 2 weeks	Task-fMRI (Amygdala Reactivity)	Amygdala reactivity to emotional faces decreased after both Ketamine and ECT.
Luo (T) et al.	2024	N = 129	Double-blind, placebo-controlled	Low (0.2 mg/kg) vs High (0.5 mg/kg)	Single bolus during anesthesia	Postoperative cognitive/emotional scales	Low dose (0.2 mg/kg) reduced postoperative depression scores without cognitive side effects; High dose increased adverse events.
Ricci et al.	2011	N = 28 (17 users, 11 controls)	Observational	Frequency:>4 times/week	Avg 4.7 years use	Serum Neurotrophins (BDNF, NGF)	BDNF levels were significantly higher in ketamine users; NGF levels were unchanged.
Scheidegger et al.	2012	N = 19	Double-blind, crossover	0.25 mg/kg	Single infusion (45 min)	rs-fMRI (Functional Connectivity)	Reduced FC between the posterior cingulate cortex (DMN) and dorsal nexus/prefrontal cortex 24h post-infusion.
Spurny-Dworak et al.	2025	N = 26	Double-blind, cross-over	56 mg	Single dose	Structural MRI (Thalamic Nuclei)	Increased volume in right thalamus (pulvinar anterior and mediodorsal nuclei) immediately after administration.
Stippl et al.	2021	N = 55 (16 fMRI)	Naturalistic / Open-label	0.5 mg/kg (rac) or 0.25 mg/kg (S-ket)	Single infusion	fMRI (Working Memory Task)	Ketamine specifically reduced cognitive symptoms; baseline DMPFC activity predicted cognitive improvement.
Tang et al.	2024	N = 264 (95 users, 169 controls)	Retrospective observational	Avg duration 65.4 months	Long-term abuse	Structural MRI (Cortical Thickness)	Widespread cortical thinning in frontal/parietal lobes; degree of thinning correlated with total lifetime consumption.
Taraku et al.	2024	N = 113 (58 TRD, 55 HC)	Longitudinal (Pre vs Post)	0.5 mg/kg	4 infusions over 2 weeks	rs-fMRI (Habenula/NAc connectivity)	Increased Habenula-visual cortex FC; decreased Habenula-parietal FC. Changes correlated with mood improvement.
Wong et al.	2016	N = 13	Single-blind	Bolus + Infusion (target 100ng/ml)	Acute (during scan)	rs-fMRI (sgACC connectivity)	Increased sgACC connectivity with hippocampus/brainstem; decreased connectivity with visual cortex.
Zavaliangos-Petropulu et al.	2023	N = 98 (66 TRD, 32 HC)	Longitudinal	0.5 mg/kg	4 infusions over 2 weeks	Structural MRI (Hippocampal Subfields)	No significant volume change in subfields over treatment. Smaller pre-treatment CA4 volumes predicted processing speed improvement.

**Table 2:** OXFORD Center for Evidence-Based Medicine (CEBM) Levels of Evidence for included Human Studies

Study	Design	Population	Grade
Luo (T) et al. (2024)	RCT, Double-blind, Placebo-controlled	129	2
Chen et al. (2020)	RCT, Double-blind, Placebo-controlled	48	2
Li, W.-C. et al. (2023)	RCT, Double-blind, Active-controlled	48	2
Liebe et al. (2018)	RCT, Double-blind, Placebo-controlled	59	2
Spurny-Dworak et al. (2025)	RCT, Double-blind, Cross-over	26	2
Scheidegger et al. (2012)	RCT, Double-blind, Cross-over	19	3
Wong et al. (2016)	Cross section	13	5
Liu (X) et al. (2025) [JAD]	Longitudinal Case-Control Cohort	40	4
Liu (X) et al. (2025) [Neuro]	Longitudinal Cohort	29	4
Taraku et al. (2024)	Clinical Trial, longitudinal	113	4
Zavaliangos-Petropulu (2023)	Clinical Trial, longitudinal	98	4
Stippl et al. (2021)	Clinical Trial, Open-label	55	4
Loureiro et al. (2020)	Cohort, Longitudinal	76	4
Curran & Morgan (2000)	Observational Cohort	39	4
Guo et al. (2022)	Retrospective Surveillance	> 5M	4
Hung et al. (2020)	Case-Control, Cross-sectional	53	4
Tang et al. (2024)	Case-Control, Cross-sectional	264	4
Ricci et al. (2011)	Case-Control, Cross-sectional	28	5
Krystal et al. (2003)	Retrospective Review, Within-subject	36	5

**Note**: 1 being the high-quality and 5 being the lowest quality.

**Table 3:** Animal study characteristics and neurotoxicity outcomes

Reference	Species	Developmental Stage	Type of ketamine	Dose	Frequency	Route	Duration	Histopathological/molecular Findings	Cognitive/behavioral outcomes	Mechanisms of action
Ahnaou et al. (2017)	Rat (Sprague-Dawley)	Adult	Ketamine	2.5, 5, 10 mg/kg	Acute & Chronic Daily	s.c.	4 weeks		Acute: Sleep inhibition (REM). Chronic: Reduced MMN response (auditory processing).	NMDAR antagonism; Altered gamma/alpha oscillations.
Bergosh et al. (2024)	Rat (Sprague-Dawley)	Adult	Ketamine	15 mg/kg	Repeated Daily	i.p.	7 days		Rescued CORT-induced grooming deficits and despair (FST).	Modulation of theta/gamma power;
Chen et al. (2021)	Mouse (ICR)	Adolescent	Ketamine	20 mg/kg	Repeated 2x daily	i.p.	14 days	Reduced PSD-95 expression in hippocampus.	Impaired social novelty; Recognition memory deficits.	Reduced fEPSP slope and LTP; NMDAR hypofunction.
Chen et al. (2021)	Rat (Sprague-Dawley)	Adult	Ketamine	10 mg/kg	Single	i.p.	Acute		Reversed depressive-like behavior induced by HDB cholinergic lesion.	Interaction with cholinergic system.
Chen et al. (2021)	Mouse (C57BL/6)	Adult	Ketamine	30 mg/kg	Single	i.p.	Acute		Induced manic-like behavior (hyperlocomotion); Reduced Novel Object Recognition preference.	Altered Ca2 + activity in TPC and PFC.
Clarke et al. (2016)	Mouse (CD1)	Adult	Ketamine	5, 10 mg/kg	Single & Repeated (3x)	i.p.	2 weeks	Increased DCX+ cells (neurogenesis) in DG; Reduced LPS-induced IL-1β and TNF-α.	Reduced immobility (FST) sustained for 8 days.	Anti-inflammatory, Promotion of hippocampal neurogenesis.
Coronel-Oliveros & Pacheco (2017)	Rat (Sprague-Dawley)	Prenatal (GD 14)	Ketamine	60 + 20 mg/kg	Single Maternal	i.m.	3 hours	Reduced CA3 layer thickness in offspring.	Offspring: Juvenile hyperactivity; Adult social withdrawal, anxiety.	Disruption of neurodevelopment; NMDAR blockade.
Daniels et al. (2023)	Rat (Wistar Kyoto)	Adult	Ketamine	10 mg/kg	Single	i.p.	Acute			Normalized 5-HT and NE neuronal firing.
De Carvalho Cartagenes (2019)	Rat (Wistar)	Adolescent	Ketamine	10 mg/kg	Repeated Daily	i.p.	3 days	Increased nitrite and lipid peroxidation (MDA).	Withdrawal: Anxiety (EPM), Depression (FST), Memory deficit.	Oxidative stress, Hippocampal damage.
Deane et al. (2020)	Gerbil (Mongolian)	Adult	Ketamine	15 mg/kg/h	Continuous	s.c.	Acute			Increased cortical gain, Stronger laminar activation.
Duan et al. (2013)	Rat (Sprague-Dawley)	Adult	Ketamine	30 mg/kg	Single	i.p.	Acute		Impaired spatial memory retrieval (MWM); Hyperlocomotion.	Blocked LTP induction; D1/D5 receptor activation.
Edem et al. (2023)	Mouse (BALB/c)	Adult	Ketamine	10 mg/kg	Repeated 4 doses	i.p.	2 weeks	Rescued CUMS-induced neuronal density loss in DG/CA1.	Rescued anxiety (EPM) and memory (Y-maze, NOR).	Modulation of nitro-oxidative stress; Oxytocin signaling.
Flowers et al. (2025)	Mouse (C57BL/6J)	Adult	Ketamine	5, 10 mg/kg	Single	i.p.	Acute	Upregulation of GluA1-containing CP-AMPARs.	Reversed CRS-induced social dysfunction and fear memory loss.	Expression of Ca2+-permeable AMPARs.
Fortress et al. (2018)	Rat (Wistar-Kyoto)	Adult	Ketamine	5 mg/kg	Repeated 2 doses	i.p.	1 week		Facilitated extinction of avoidance behavior.	Enhanced population spike LTP.
Gass et al. (2018)	Rat (Sprague-Dawley)	Adult	S-Ketamine	10 mg/kg	Single	s.c.	Acute		Resting state fMRI	Reduced global clustering coefficient; Increased connectivity in reward circuitry.
Gass et al. (2019)	Rat (Sprague-Dawley)	Adult	S-Ketamine	10 mg/kg	Single	s.c.	Acute		Behavioral escape deficit in NC strain.	Modulation of prefrontal strength and segregation.
Goswamee et al. (2022)	Mouse (C57BL/6)	Adult	Ketamine	10, 32 mg/kg	Single vs Repeated	s.c.	4 weeks		Spatial working memory deficit (Y-maze).	Increased mPFC-driven firing in Nucleus Reuniens.
Hajizadeh Moghaddam et al. (2024)	Mouse (Male)	Adult	Ketamine	20 mg/kg	Single	i.p.	Acute	Increased MDA; Decreased CAT, SOD, GSH, TAC (Oxidative stress).	Depressive-like (FST) and anxiety-like (OFT) behaviors; Memory impairment (NORT).	Oxidative stress; Dopaminergic dysregulation.
Hong et al. (2022)	Mouse (C57BL/6)	Adolescent (P21)	Ketamine	30 mg/kg	Repeated (3X/day)	i.p.	7 days	Increased IL-1 β, IL-6; Increased Tau/p-Tau/Aβ.	Cognitive impairment (MWM); Reduced exploration (OFT).	Neuroinflammation; Tau hyperphosphorylation.
Hsieh et al. (2021)	Mouse (ICR)	Adolescent	Ketamine	20 mg/kg	Repeated (2x daily)	i.p.	14 days		Recognition memory deficits; Social withdrawal.	Reduced basal synaptic transmission and LTP.
Huang et al. (2016)	Mouse (C57BL/6)	Adult	Ketamine	1–30 μM	In vitro	Bath	Acute			Selectively inhibited LTD (1–10 μM); Protected gamma oscillations.
Iafrati et al. (2014)	Mouse (Heterozygous Reeler)	Juvenile	Ketamine	30, 100 mg/kg	Single	i.p.	Acute	Increased spine density in PFC.	Restored fear memory renewal.	mTOR signaling pathway activation.
Izumi & Zorumski (2014)	Rat (Sprague-Dawley)	Adolescent	Ketamine	1–20 μM	In vitro	Bath	Acute			Inhibition of NMDAR-LTP induction.
Jordan et al. (2023)	Rat (Sprague-Dawley)	Adult	Ketamine	12.5 mg/kg	Single	i.v.	40 min	No microscopic abnormalities (Olney's lesions).	No clinical signs of neurotoxicity.	
Khan et al. (2018)	Rat (Sprague-Dawley)	Adult	Ketamine	10, 17, 30 mg/kg	Single	p.o.	Acute		Drug discrimination: NYX-2925 does not substitute for ketamine.	NMDA receptor modulation.
Li et al. (2019)	Rat (Sprague-Dawley)	Adolescent	Ketamine	20, 30 mg/kg	Repeated Daily	i.p.	21 days	Increased TUNEL+ cells (apoptosis) in PFC.	Depressive-like behavior (FST) when combined with ethanol.	Apoptosis via mitochondrial pathway.
Logue et al. (2022)	Rat (Sprague-Dawley)	Adult	Ketamine	2.5, 5 mg/kg	Single	i.p.	Acute		Rescued isolation-induced LTP deficits (Males).	Sex-specific modulation of hippocampal plasticity.
Luo et al. (2021)	Mouse (C57BL/6)	Adult	Ketamine	30 mg/kg	Repeated Daily	i.p.	28 days	Reduced dendritic spine density; Reduced PSD-95, Synapsin.	Impaired spatial learning/memory (MWM).	Restraint of CaMKII-ERK-CREB signaling.
Miao et al. (2023)	Rat (Sprague-Dawley)	Adult	Ketamine	50 mg/kg	Repeated Daily	i.p.	7 days	Reduced Nissl+ neurons in LC; Increased CaMKII/p-CREB.	Impaired spatial learning (MWM).	Neurotoxicity in LC; CaMKII/CREB upregulation.
Michaelsson et al. (2019)	Rat (Wistar)	Adolescent & Adult	Ketamine	10 mg/kg	Single	i.p.	Acute	Increased Ki-67 + cells in Dentate Gyrus.	Reversed anhedonia (SPT).	Enhanced neurogenesis.
Mingardi et al. (2023)	Rat (Sprague-Dawley)	Adult	Ketamine	10 mg/kg	Single	i.p.	Acute	Rescued stress-induced dendritic retraction in PFC.	Rescued anhedonia (SPT).	Restoration of glutamate transmission.
Musat et al. (2025)	Mouse (C57BL/6N)	Young & Aged	Ketamine	50 mg/kg	Single	i.p.	Acute	No effect on liver injury severity; Altered GFAP/Iba1 signals.	Relieved anhedonia/anxiety; Improved sociability (aged).	Anti-inflammatory; Age-dependent effects.
Patton et al. (2017)	Rat (Sprague-Dawley)	Adult	Ketamine	10 mg/kg	Single	i.p.	Acute	Increased p-JAK2 and p-STAT3 in OFC.	Corrected stress-induced reversal learning deficit.	JAK2/STAT3 signaling pathway activation.
Piazza et al. (2024)	Mouse (C57BL/6)	Adult	Ketamine	5 mg/kg	Single	i.p.	Acute		No impairment of LTP induction.	Expression of Ca2 + permeable AMPARs.
Riggs et al. (2025)	Rat (Wistar-Kyoto)	Adult	(2R,6R)-HNK	10 mg/kg	Single	i.p.	Acute		Improved spatial novelty detection.	Restored LTP in hippocampus.
Sala et al. (2022)	Rat (Sprague-Dawley)	Adult	Ketamine	10 mg/kg	Single	i.p.	Acute	Rescued stress-induced dendritic retraction in PFC.	Rescued anhedonia (SPT) and fear extinction.	Normalization of glutamate release.
Staszelis et al. (2022)	Rat (Sprague-Dawley)	Adult	Ketamine	10 mg/kg	Single	s.c.	Acute		Psychotic-like behaviors.	Elevation of gamma power; Disinhibition.
Stewart & Reid (1994)	Rat (Hooded Lister)	Adult	Ketamine	10 mg/kg	Repeated (with ECS)	i.p.	20 days		Reduced seizure duration; Prevented ECS-induced synaptic enhancement.	NMDAR blockade during seizure.
Sun et al. (2014)	Monkey (Cynomolgus)	Adolescent	Ketamine	1 mg/kg	Repeated Daily	i.v.	6 months	Increased TUNEL+ cells (apoptosis) in PFC.	Decreased locomotor activity.	Apoptotic pathway activation.
Tian et al. (2025)	Mouse (ICR)	Adult	S-Ketamine	10 mg/kg	Single	i.p.	Acute	Improved dendritic spine density; Reduced NLRP3.	Alleviated depressive-like behaviors.	Anti-inflammatory; BDNF-TrkB signaling.
Vecchia et al. (2021)	Rat (Wistar)	Adult	Ketamine	5, 10, 15 mg/kg	Repeated Weekly	i.p.	4 weeks	No effect on 6-OHDA induced TH loss.	Reversed anhedonia and social memory deficits.	Antidepressant-like effects.
Wan et al. (2024)	Mouse (C57BL/6J)	Neonatal (P7)	Ketamine	20 mg/kg	Repeated (3x/day)	i.p.	3 days	Decreased dendritic spine density in CA1; Reduced BDNF.	Cognitive impairment (MWM, NORT) in adulthood.	BDNF downregulation; Impaired synaptic plasticity.
Wen et al. (2024)	Rat (Sprague-Dawley)	Aged	Esketamine	10 mg/kg	Single	i.p.	Acute	Decreased Iba1, IL-6; Increased BDNF.	Alleviated POCD and POD.	Inhibition of Microglial M1 polarization.
Wen et al. (2024)	Mouse (C57BL/6)	Adult	Ketamine	10 mg/kg	Single	i.p.	Acute	Reduced pyroptosis markers (GSDMD, NLRP3).	Relieved depressive behaviors.	Inhibition of astrocyte pyroptosis.
Xu et al. (2023)	Mouse	Adult	(2R,6R)-HNK	30 mg/kg	Single	i.p.	Acute	Increased c-fos positive cells in BLA.	Regulates fear memory extinction.	Enhanced synaptic transmission.
Yang et al. (2018)	Mouse (C57BL/6)	Adult	Ketamine	10 mg/kg	Single	i.p.	Acute	Restored NR2B expression in hippocampus.	Reversed depressive-like behavior (SPT, Social interaction).	Modulation of NMDAR subunit expression.
Yu et al. (2012)	Monkey (Cynomolgus)	Adolescent	Ketamine	1 mg/kg	Repeated Daily	i.v.	6 months	Reduced TH+ axons in PFC.	Locomotive depression.	Reduced neural activity in VTA/SN.
Zhang et al. (2021)	Mouse (C57BL/6)	Adult	Ketamine	10 mg/kg	Single	i.p.	Acute	Synaptic ultrastructure restoration in PFC.	Improved anxiety/depression; No effect on spatial memory.	HCN1-BDNF signaling.
Zhang et al. (2023)	Mouse (C57BL/6)	Adult	S-Ketamine	10 mg/kg	Repeated Daily	i.p.	7 days	Decreased reactive astrocytes; Inhibited NLRP3.	Improved cognitive dysfunction and depression.	Inhibition of hippocampal astrocytosis.
Zhao et al. (2014)	Rat (Sprague-Dawley)	Prenatal (GD 14)	Ketamine	40 mg/kg	Single Maternal	i.v.	2 hours	Neuronal loss in CA3; Reduced BrdU+ cells.	Offspring: Anxiety, Depression, Spatial memory impairment.	Neurotoxicity; Apoptosis.
Zhou et al. (2025)	Rat (Sprague-Dawley)	Neonatal (P7)	Esketamine	25, 50 mg/kg	Repeated Daily	i.p.	3 days	White matter damage (DTI): Corpus Callosum.	High dose: Depression, Memory impairment.	White matter microstructural integrity disruption.
Goswamee et al. (2022)	Mouse (C57BL/6)	Adult	(2R,6R)-HNK	10, 32 mg/kg	Single and Repeated	s.c.	4 weeks		No cognitive deficit in Y-maze.	Increased intrinsic excitability of RE neurons.
Riggs et al. (2025)	Rat (Wistar-Kyoto)	Adult	(2R,6R)-HNK	10 mg/kg	Single	i.p.	Acute		Improved spatial novelty detection.	Restored LTP in hippocampus.
Xu et al. (2023)	Mouse	Adult	(2R,6R)-HNK	30 mg/kg	Single	i.p.	Acute	Increased c-fos in BLA.	Regulates fear memory extinction.	Enhanced synaptic transmission.
Becker & Grecksch (2004)	Rat	Adult	Ketamine	Varies	Repeated	i.p.	Chronic		Psychosis-like behavior.	

**Note**: IV, intravenous; IM, intramuscular; SC, subcutaneous; IP, intraperitoneal.

## Data Availability

Data are publicly available.
